# Development and validation of a disulfidptosis and disulfide metabolism-related risk index for predicting prognosis in lung adenocarcinoma

**DOI:** 10.1186/s12935-023-03204-1

**Published:** 2024-01-02

**Authors:** Leqi Zhong, Wuguang Chang, Bin Luo, Wuyou Gao, Huanhuan He, Mouxiang Fang, Hongmu Li, Zhesheng Wen, Youfang Chen

**Affiliations:** 1 Department of Thoracic Surgery, State Key Laboratory of Oncology in South China, Collaborative Innovation Center for Cancer Medicine, Sun Yat-sen University Cancer Center, Guangzhou, 510060 China; 2 Department of Nuclear Medicine, The Fifth Affiliated Hospital, Sun Yat-sen University, Zhuhai, 519000 China; 3 Guangdong Provincial Key Laboratory of Biomedical Imaging and Guangdong Provincial Engineering Research Center of Molecular Imaging, The Fifth Affiliated Hospital, Sun Yat-sen University, Zhuhai, 519000 China; 4 Department of Pathology, State Key Laboratory of Oncology in South China, Collaborative Innovation Center for Cancer Medicine, Sun Yat sen University Cancer Center, Guangzhou, 510060 China; 5 Institute of Biophotonics, South China Normal University, Guangzhou, China

**Keywords:** Disulfidptosis, Disulfide metabolism, TXNRD1, Lung adenocarcinoma, Immunotherapy

## Abstract

**Background:**

Disulfidptosis is a recently proposed novel cell death mode in which cells with high *SLC7A11* expression induce disulfide stress and cell death in response to glucose deficiency. The purpose of the research was to explore the function of disufidptosis and disulfide metabolism in the progression of lung adenocarcinoma (LUAD).

**Methods:**

The RNA-seq data from TCGA were divided into high/low expression group on the base of the median expression of *SLC7A11*, and the characteristic of differentially expressed disulfide metabolism-related genes. Least absolute shrinkage and selection operator (LASSO) algorithm was conducted the disulfidptosis and disulfide metabolism risk index. The tumor mutation burden (TMB), mechanism, pathways, tumor microenvironment (TME), and immunotherapy response were assessed between different risk groups. The role of *TXNRD1* in LUAD was investigated by cytological experiments.

**Results:**

We established the risk index containing 5 genes. There are significant differences between different risk groups in terms of prognosis, TMB and tumor microenvironment. Additionally, the low-risk group demonstrated a higher rate of response immunotherapy in the prediction of immunotherapy response. Experimental validation suggested that the knockdown of *TXNRD1* suppressed cell proliferation, migration, and invasion of LUAD.

**Conclusion:**

Our research highlights the enormous potential of disulfidptosis and disulfide metabolism risk index in predicting the prognosis of LUAD. And *TXNRD1* has great clinical translational ability.

**Supplementary Information:**

The online version contains supplementary material available at 10.1186/s12935-023-03204-1.

## Introduction

Lung cancer is the main reason of death from cancer [1], the 5-year survival rate is only 4 to 17 percent [2]. The majority, about 40%, of lung cancer, are lung adenocarcinoma [3]. In the past few decades, with the continuous deepening of knowledge of molecular mechanisms, drugs targeting EGFR, ALK, PD-1/PD-L1 have profoundly changed the treatment strategy of non-small cell lung cancer (NSCLC) [4, 5]. However, given the heterogeneity of the tumor in each patient, immunotherapy is ineffective in around 70% of patients with advanced NSCLC [6]. Therefore, identifying accurate and effective biomarkers is of great value for the survival of LUAD patients.

Disulfidptosis is a newly cell death discovered by Liu et al., which is independent of existing programmed cell death such as apoptosis, ferroptosis, cuproptosis, and necroptosis. The *SLC7A11* transporter, a member of the Solute Carriers (SLC) family, performs a critical function in the maintenance of intracellular glutathione levels and the protection of cells from oxidative stress-induced cell death [7]. Liu et al. found that when glucose supply was restricted, the oxidoreduction force was insufficient, leading to abnormal accumulation of cystine or other disulfide molecules in *SLC7A11*-high cells, finally inducing disulfide stress to trigger cell death [8]. And the reducing agent of disulfide stress can completely inhibit this death process. In addition, this study found that glucose transporter 1 inhibitors can effectively inhibit cell glucose uptake, thereby inducing disulfide death in *SLC7A11* overexpressing cells; the inhibitor also has a significant effect on tumor growth with high expression of *SLC7A11* in mice. The discovery of the mechanism of disulfidptosis provides a new framework for targeted cancer therapy.

In this study, disulfide metabolism-related genes were characterized in *SLC7A11* samples with different expressions and a robust risk index was constructed for LUAD patients. Cell experiments have confirmed that *TXNRD1* has the potential to become a target for LUAD treatment.

## Material and methods

### Data collection and processing

**Fig. 1:**
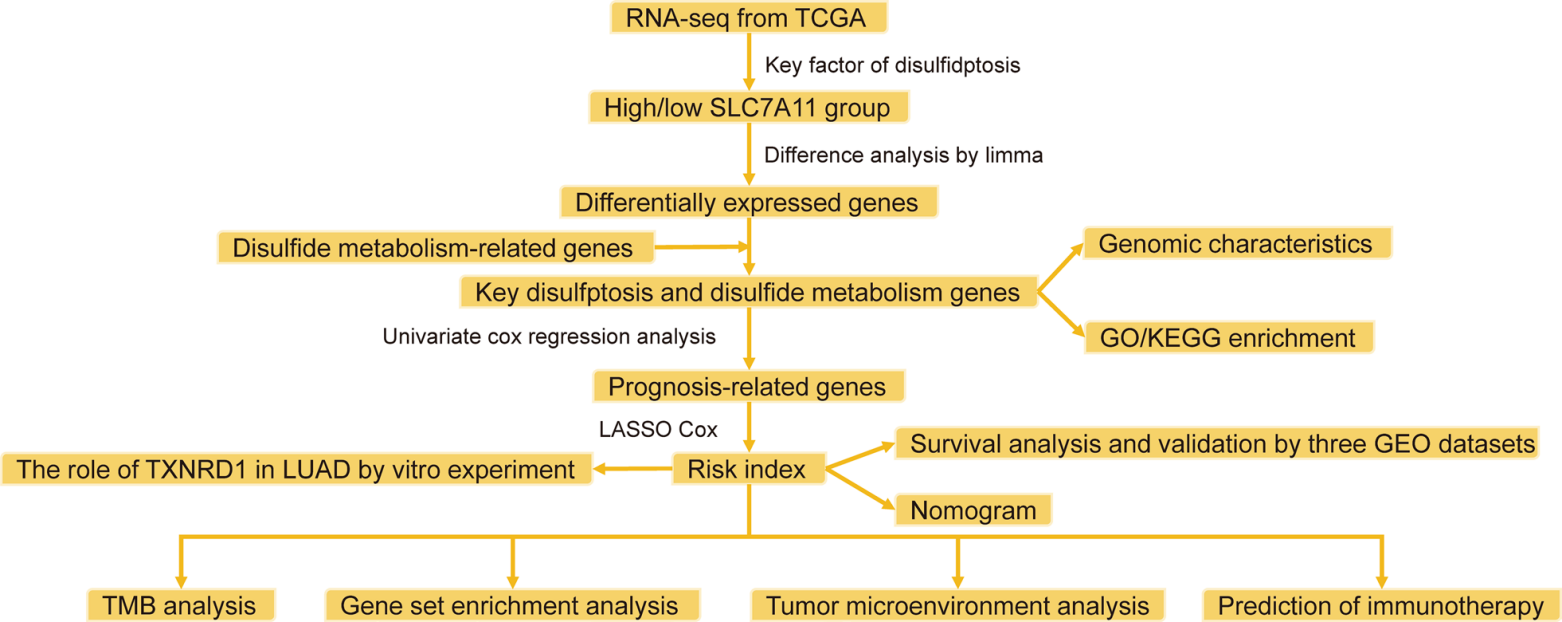
Flowchart in this study

The flowchart of this study was shown in Fig. 1. The RNA-seq data, clinical information, and somatic mutation data were downloaded from the TCGA database (https://portal.gdc.cancer.gov/). After excluding cases with missing survival information or survival time was less than 30 days, 485 LUAD cases were included in the training cohort. The count format was employed for difference analysis, and log2(TPM + 1) conversion was used for subsequent analysis. Three LUAD dataset were downloaded from GEO (https://www.ncbi.nlm.nih.gov/geo/), including GSE72094 (n = 398), GSE68465 (n = 418) and GSE37745 (n = 226) as validation cohorts, and we performed the necessary log2 conversion. We obtained genes with correlation scores in the top 200 from GeneCards (https://www.genecards.org/) [9].

### Identification the characteristics of disulfide metabolism-related genes

According to the median expression of *SLC7A11*, TCGA cohort was divided into *SLC7A11*-high or low group. Then ‘limma’ R package [10] was used for differential analysis to select differentially expressed disulfide metabolism-related genes. The standards of differentially expressed genes (DEGs) were defined as false discovery rate (FDR) < 0.05 and log2 |fold change|≥ 1. Gene Ontology (GO) and Kyoto Encyclopedia of Genes and Genomes (KEGG) enrichment analysis was performed used the ‘clusterProfiler’ R package [11].

### Construction of the risk index

Univariate cox regression analysis was used to preliminarily screen survival-related genes (p < 0.05), and then LASSO cox regression was used to obtain the final candidate genes. The coefficient of each candidate gene multiplied by the expression yielded a risk index for each patient. In accordance with the median risk index, LUAD patients were divided into high/low -risk group for subsequent analysis. The difference in survival between the two groups was compared by Kaplan–Meier (KM) curve and log-rank test. The same method was used to calculate the risk index in GSE72094, GSE68465, and GSE37745 to verify the accuracy of the risk index.

### Development and validation of the nomogram

To find out whether the risk index was independent of typical clinical parameters, univariate and multivariate cox regression analyses were carried out. The nomogram was constructed based on the risk index and risk factors (p < 0.05). Calibration curves, decision curve analysis (DCA) and time-dependent receiver operating characteristic (ROC) curves at 1, 3, and 5 years were used to evaluate the prognostic value of the nomogram.

### Association with TMB

Somatic mutation data for LUAD patients were downloaded from the TCGA database and Based on the total number of somatic mutations per MB of exon coding region in the human genome, we calculated the TMB for each sample as the total number of somatic mutations/35 MB. The ‘maftools’ R package [12] was used to visualize the somatic mutation of high and low risk score groups, and the correlation between risk score and TMB was calculated. The ‘somaticInteractions’ function utilized paired Fisher’s exact test to analyze the co-occurrence and exclusiveness among the top 20 with mutation incidence.

### Gene set enrichment analysis

To explore the differences in biological characteristics and pathways between different subgroups, we downloaded ‘c5.go.v7.5.1.symbols’ and ‘c2.cp.kegg.v7.5.1.symbols’ from the Molecular Signature Database for executing GSEA software (version: 4.2.3) [13].

### Analysis of tumor microenvironment

The ssGSEA algorithm was used to calculate the proportion of 28 immune cells in the tumor microenvironment [14]. ESTIMATE algorithm computes tumor purity and tumor microenvironment score of each patient (including ESTIMATE score, immune score, stromal score and tumor purity) [15]. Tumor Immune Single-cell Hub (TISCH) database is an online analysis website that integrates various cancer single-cell sequencing data (http://tisch.comp-genomics.org/) [16]. We used GSE131907 in the database to explore the characteristics of the tumor microenvironment of key genes at the single-cell level, which contains single cell sequencing data of 40 primary or metastatic LUAD.

### Prediction of immunotherapy response

An online analytic tool called Tumor Immune Dysfunction and Exclusion (TIDE) model tumor immune escape to forecast the effectiveness of cancer immunotherapy [17]. We computed the TIDE score of each patient based on the expression profile of LUAD, and a higher TIDE score means that immune escape is more likely to occur during immunotherapy. We also downloaded the immunotherapy dataset (Checkmate 009/010/025) for renal clear cell carcinoma to evaluate the predictive performance of the riskscore, which includes 181 samples of advanced renal clear cell carcinoma who treated with anti-PD-1 monoclonal antibody [18].

### Cell culture and siRNA transfection

Two human-derived NSCLC cell lines (A549 and HCC44), frozen and stored in liquid nitrogen after purchased from the Xinyuan Biotech Co. Ltd. (Shanghai, China), were applied in this research. Authentication of cell lines was performed by short tandem repeat (STR) analysis. Cells were checked routinely and no bacterial, mycoplasma or fungal contamination was confirmed. Cells were cultured in RPMI-1640 medium (Gibco, Grand Island, NY, USA) containing 10% fetal bovine serum (Sinsage, Beijing, China). Cells were maintained in a humidified atmosphere of 5% CO_2_ at 37 °C. si-TXNRD1s and negative control (siRNA-nc) were commercially synthesized (Suzhou Gene Pharma, China). The selected sequences of small interfering RNAs (siRNAs) were as follows:

si-TXNRD1-1: 5′-GCCAUGGUCCAACCUUGAATT-3′,

si-TXNRD1-2: 5′-GGAGCAUCCUAUGUCGCUUTT-3′,

si-TXNRD1-3: 5′-CCACUGUAUUUACUCCUUUTT-3′,

si-NC: 5′-UUCUCCGAACGUGUCACGUTT-3′. The siRNA oligos were transfected into A549 and HCC44 cells with Lipofectamine 3000 (Invitrogen, USA) according to the manufacturer’s instructions.

### Western blotting

Standard procedures were employed to do the Western blotting. Briefly, the cell lysates were prepared using RIPA lysis buffer supplemented with protease inhibitor cocktail, phosphatase inhibitor cocktail, and 1 mM PMSF (all from Sigma-Aldrich). Afterwards, samples were quantified using Bradford method. Same amount of protein was separated on 7.5–12% Bis–Tris gels (Epizyme, Shanghai, China) using MOPS/MES buffer. The electrophoretically isolated proteins were then transferred to polyvinylidene fluoride (PVDF) membranes (Invitrogen, California, USA). Membranes were blocked for 90 min at 4 °C with 5% non-fat milk in PBST (0.1% Tween 20) and incubated overnight at 4 °C in solution containing relevant primary antibodies: mouse anti-GAPDH (Proteintech, Chicago, USA) 1:10000 and rabbit anti-TXNRD1 (Proteintech, Chicago, USA) 1:3000. Subsequently, horseradish peroxidase (HRP)-conjugated secondary antibody was diluted with PBST containing 3% non-fat milk and applied to the blots for an hour at room temperature. The ECL Chemiluminescence System (Tianneng, Shanghai, China) was implemented to examine antibody binding and analyzed using Image J software (version 2.0, LOCI, University of Wisconsin, Madison, WI, USA).

### Viability and proliferation testing

Cell viability was assessed using Cell Counting Kit-8 (CCK-8) (Beyotime Biotechnology, Shanghai, China). Cells were cultured in 96-wells plates (1000 cells per well). After incubation, CCK-8 solution was added to each well followed by a further 2 h incubation under 5% CO_2_ at 37 °C. Absorbance was automatically measured at 450 nm using a microplate reader (Infinite F50, Tecan Group Ltd., Mannedorf, Switzerland) in day 1 to day 6. Cell proliferation was assessed using plate clone formation and 5-ethynyl-2′-deoxyuridine (EdU) assays. Cells were cultured in 12-wells plates (1000 cells per well) after the transfection of siRNA oligos, and the culture medium was refreshed every 3 days. Cell colonies were fixed and then stained with gentian violet (Shanghai yuanye Bio-Technology Co., Ltd, China) after 7 days. The EdU assay was managed according to product instructions (Epizyme, Shanghai, China). The Edu-positive rate was computed as EdU-positive cells/Hoechst-stained cells × 100%.

### Migration and invasion testing

Transwell (Corning, NY, USA) assays for cell migration and invasion were used to measure these processes. For 48 h, cells were precultured in media devoid of serum. RPMI-1640 with 10% FBS was placed in the lower chamber, and 3 104 cells were put to serum-free media in the top chamber for the migration test. After 36 h, the non-migrating cells on the upper chambers were carefully cleaned using a cotton swab, and the migrating cells on the bottom of the filter were stained and counted.

Cell migration and invasion were measured by Transwell (Corning, NY, USA) migration and invasion assays. Cells were precultured in serum-free medium for 48 h. For migration assay, 3 × 10^4^ cells were added to serum-free medium in the upper chamber, and the lower chamber was filled with RPMI-1640 containing 10% FBS. The non-migrating cells on the upper chambers were gently removed with a cotton swab after 36 h, and the migrated cells on the filter's underside were stained and counted. Transwell inserts (Corning, NY, USA) coated with Matrigel/fibronectin (BD Biosciences, NY, USA) were used to conduct Matrigel invasion experiments. For each plate, five distinct fields were recorded. Each experimental process was carried out in triplicate.

### Statistical analysis

Data analysis and image production were conducted using R software (version 4.1.3) and Graphpad Prism 9. Cell counting of Edu assay, migration and invasion assays were performed using Image J software (version 2.0, LOCI, University of Wisconsin, Madison, WI, USA). For the bioinformatics analysis section, for comparisons between continuous variables, the Wilcoxon test was employed, while the chi-square test was utilized for comparisons between categorical variables. Survival analysis was performed by log-rank test. The ‘regplot’ R package is used to plot nomogram. Spearman rank correlation was used for correlation analysis. For the in vitro experiments analysis part, data were expressed as mean ± standard deviations (SD). The statistical significance between groups was assessed by analysis of variance (ANOVA) with Sidak’s multiple comparisons test. Differences were considered to be statistically significant when the P value was 0.05 or less.

## Results

### Identification of disulfide metabolism-related genes

**Fig. 2:**
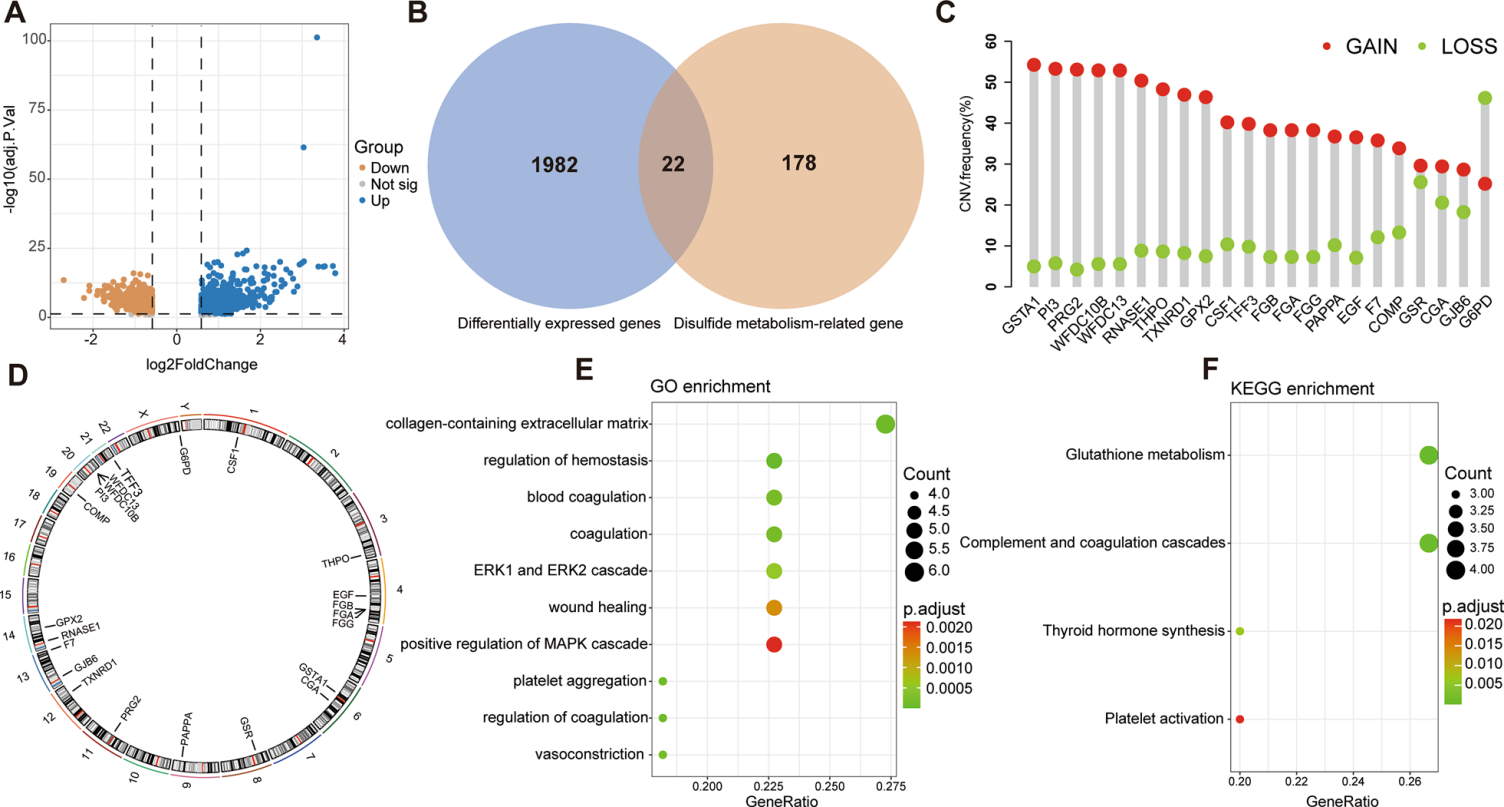
Genomic characteristic and enrichment analysis of disulfidptosis and disulfide metabolism-related genes. **A** Volcano plot exhibiting down or low- regulated genes. **B** Venn diagram showed 22 differentially expressed disulfide metabolism-related genes. **C** The CNV mutation frequency of 22 key genes. **D** Chromosome position of disulfide metabolism-related genes. **E** GO enrichment analysis. **F** KEGG enrichment analysis

After difference analysis of different *SLC7A11* expression groups, 2004 genes were obtained (Fig. 2A), and 22 candidate genes were obtained after intersection with disulfide metabolism-related genes (Fig. 2B). By analyzing CNV of 22 candidate genes, we found that except for G6PD, the other 22 genes had a higher percentage of CNV gain (Fig. 2C). The positions of 22 genes on chromosomes are shown in Fig. 2D. GO and KEGG enrichment analysis results indicated that the function of the above genes was mainly concentrated in the coagulation process, wound healing, and glutathione metabolism (Fig. 2E, F). Disulfide bonds are covalent bonds formed through redox reactions, and their presence can increase the stability and structural strength of proteins. Many drugs that promote wound healing are linked by disulfide bonds, and when they enter the corresponding wound site, they are cleaved by glutathione, releasing drugs that promote wound healing [19, 20]. This is consistent with the results of enrichment analysis.

### Construction and validation of a disulfptosis and disulfide metabolism risk index

**Fig. 3:**
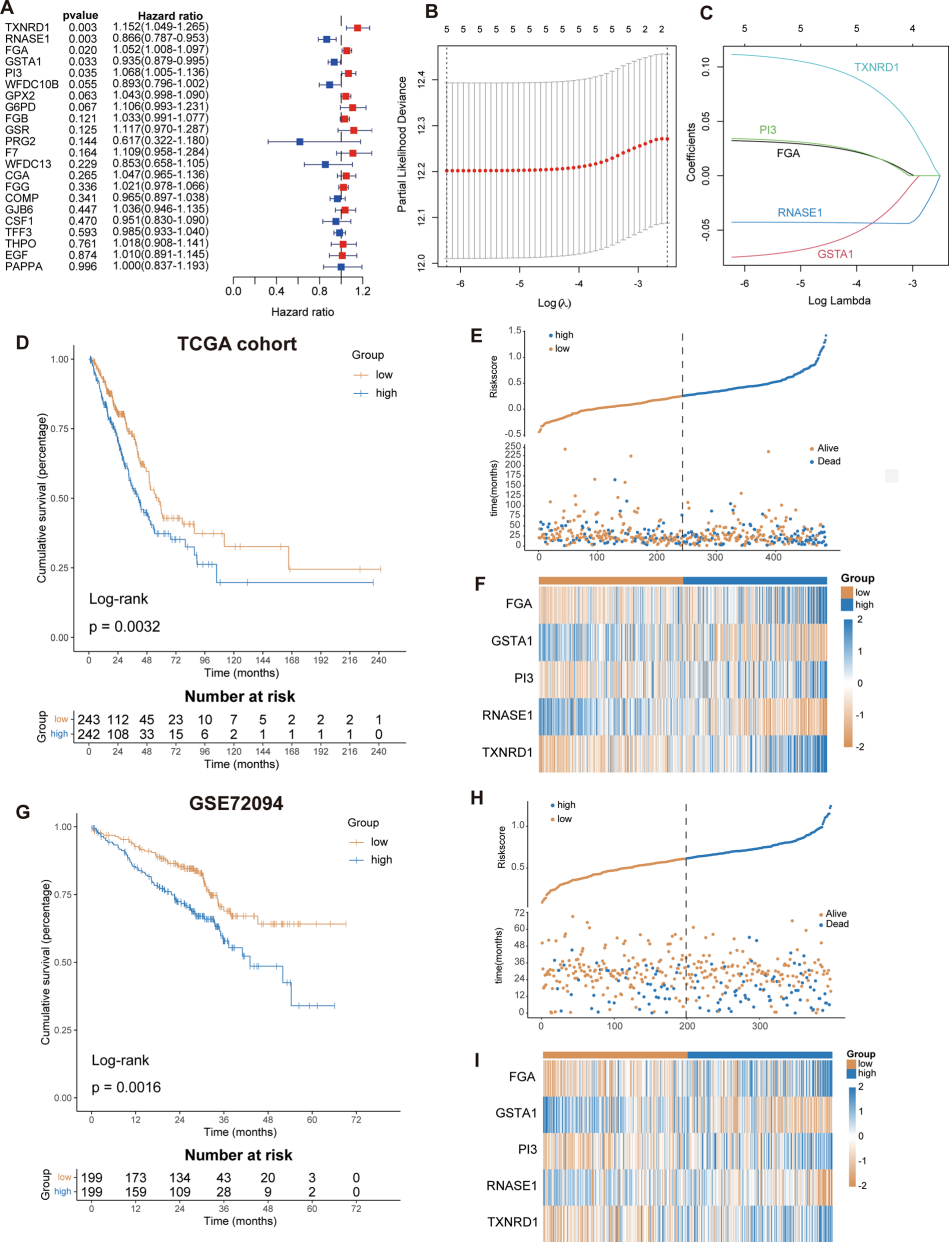
Construction and validation of disulfidptosis and disulfide metabolism-related risk index. **A** Univariate cox analysis of 22 key genes. **B** Adjustment of parameters (lambda) in the LASSO Cox regression model using tenfold cross-validation. **C** 5 key disulfide metabolism-related genes and their coefficients. **D** KM survival analysis in TCGA dataset. **E** Changes in the number of deaths as the risk score increases in TCGA dataset. **F** Heatmap showing the expression in TCGA dataset. **G** KM survival analysis in GSE72094 dataset. **H** Changes in the number of deaths as the risk score increases in GSE72094 dataset. **I** Heatmap showing the expression in GSE72094 dataset

Univariate cox regression analysis found that 5 of the 22 genes were significantly associated with prognosis (Fig. 3A). These five genes were then incorporated into the lasso regression model. When lambda value was optimal, we found that all the 5 genes were included in lasso model (Fig. 3B, C), then we got risk index of each patient: *FGA*exp*0.03223269−*GSTA1*exp*0.07487323 + *PI3*exp*0.03419497−*RNASE1*exp*0.04274036 + *TXNRD1*exp*0.11154829. Patients with TCGA-LUAD were divided into two groups based on the median risk score. Survival analysis results showed significant differences in OS between the two groups (Fig. 3D). Risk factor distribution and heatmap reflect the rationality of risk index (Fig. 3E, F). Risk indexes were calculated in the same way for each sample in the independent dataset GSE72094, and survival analysis showed that the low-risk group had a worse prognosis (Fig. 3G). Similar results were obtained from risk factor distribution and heatmaps (Fig. 3H, I). In addition, the other two GEO datasets (GSE68465 and GSE37745) also obtained similar KM curves and risk factor graphs (Additional file 1: Fig. S1A–F).

### Development of the nomogram

**Fig. 4:**
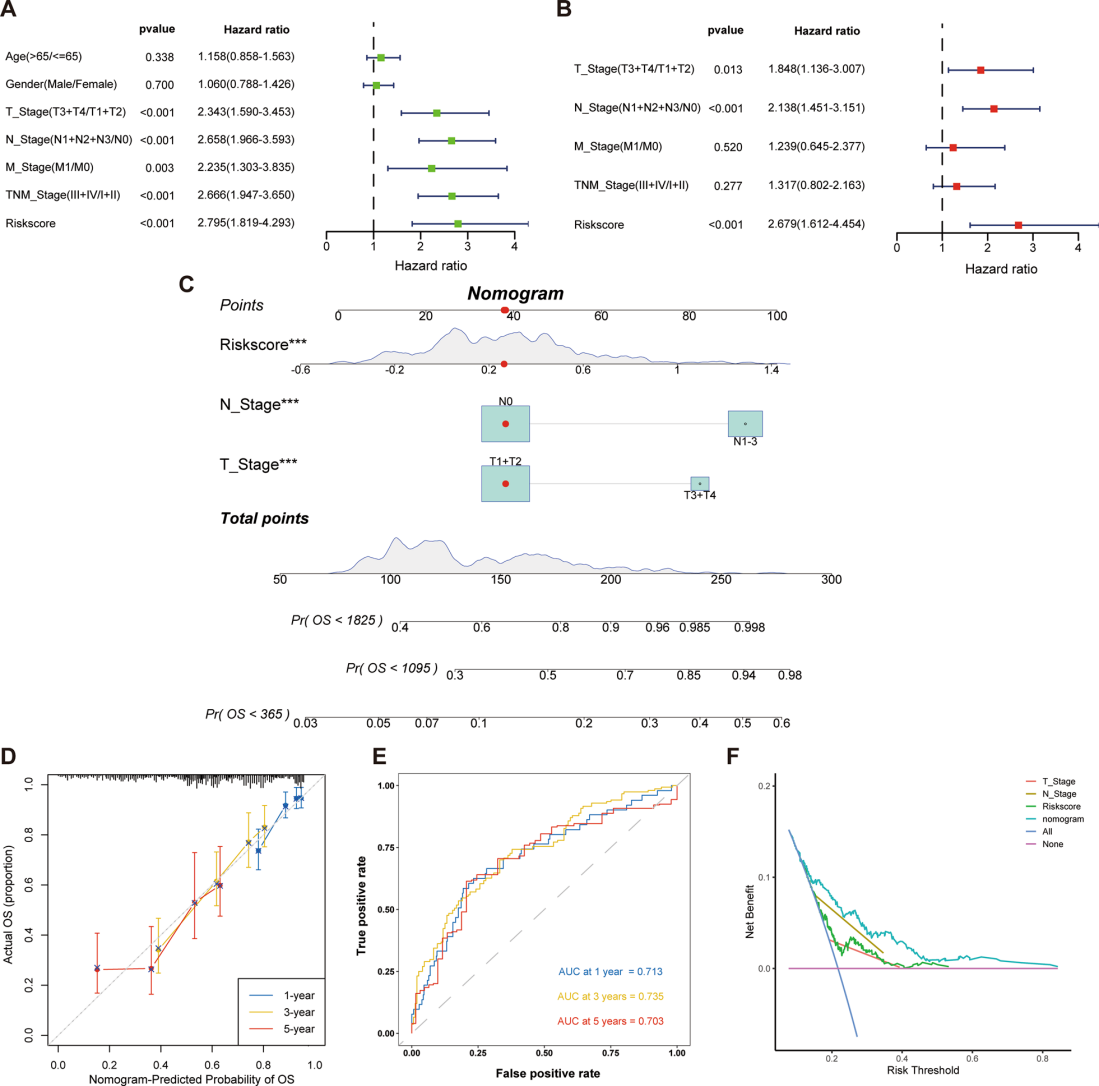
Development of the nomogram to predict the prognosis of LUAD. Univariate (**A**) and multivariate (**B**) Cox regression analysis of risk score. **C** Nomogram for the prediction of 1-, 3- and 5-year survival probability. **D** Time-dependent ROC analysis of the nomogram. **E** Calibration curves for evaluating the accuracy. **F** DCA demonstrated the degree of benefit of different factors. ***p < 0.001

In order to determine if the risk index represented a separate risk factor for the prognosis of LUAD, univariate and multivariate cox regression analyses were used (Fig. 4A, B). The results showed that risk index was a negative risk factor for OS (HR = 2.795, 95%CI 1.819–4.293, p < 0.0001). Next, a column chart was constructed based on the risk index, T staging and N staging to predict OS in years 1, 3 and 5 (Fig. 4C). The actual results and the projected outcomes are essentially consistent, according to calibration curves (Fig. 4D). The time-dependent ROC curve also showed better predictive power of the nomogram (Fig. 4E). The DCA curve indicated that nomogram had higher benefits compared to other single factors (Fig. 4F).

### Analysis of tumor mutation burden

**Fig. 5:**
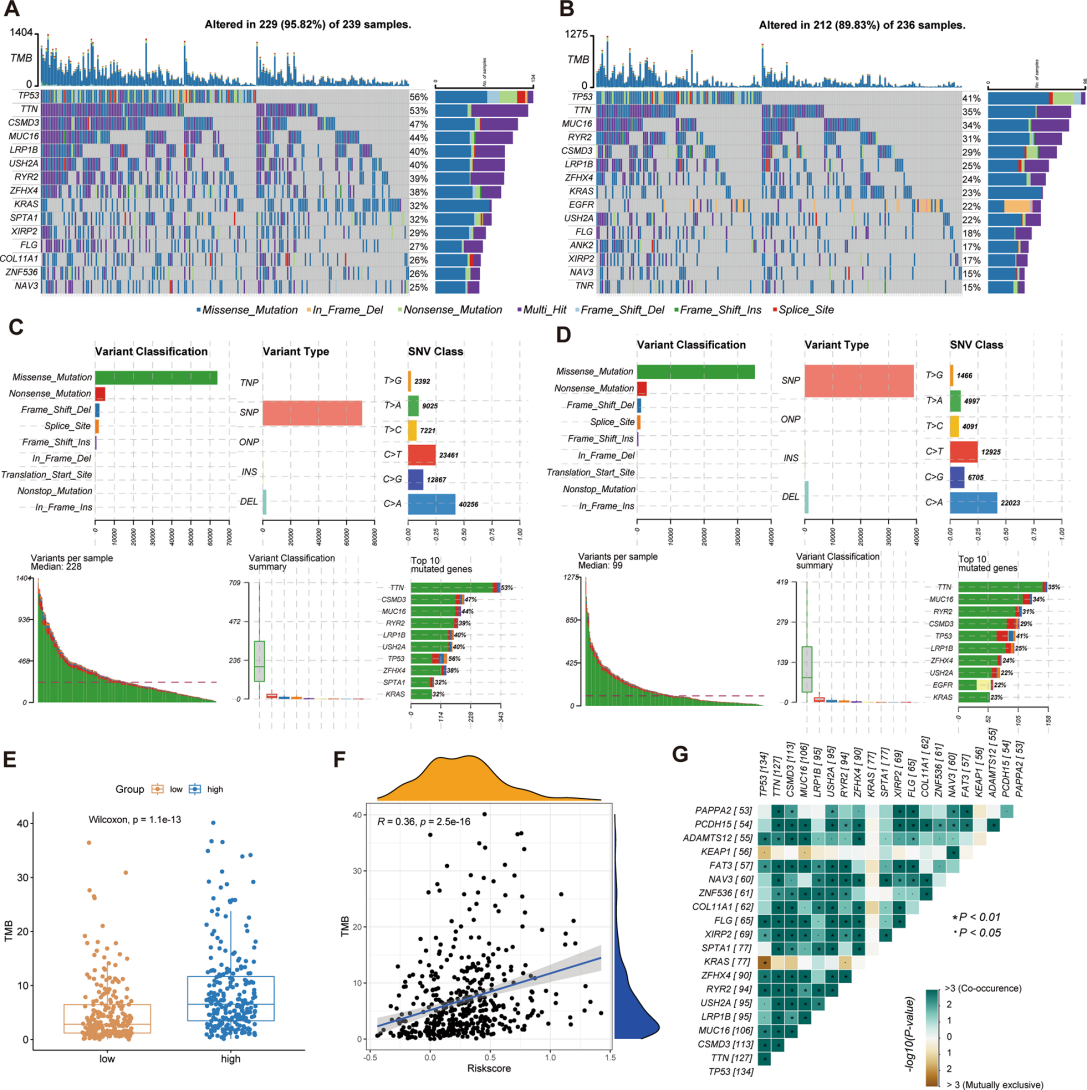
Analysis of TMB in LUAD. Genes with the top 15 mutation frequencies in high-risk group (**A**) and low-risk group (**B**). Detailed mutation type statistics in high-risk group (**C**) and low-risk group (**D**). **E** Statistical analysis of TMB between two risk groups. **F** Correlation analysis between risk score and TMB. **G** Fisher’s Exact test to detect mutually exclusive or co-occuring events

We calculated the TMB for each LUAD patient and showed the difference in TMB between the high-risk group (Fig. 5A) and the low-risk group (Fig. 5B) using the waterfall plot. Figure 5C, D displays comprehensive mutation statistic. 9 of the top 10 genes in the two subgroups overlapped, but the high-risk group experienced more mutations than the low-risk group did. On the whole, TMB in high-risk group was higher than that in low-risk group (Fig. 5E), and TMB was positively correlated with risk index (Fig. 5F). According to the analysis of the top 20 genes with mutation incidence, co-occurrence was found among most of the gene mutations (Fig. 5G).

### Gene set enrichment analysis

**Fig. 6:**
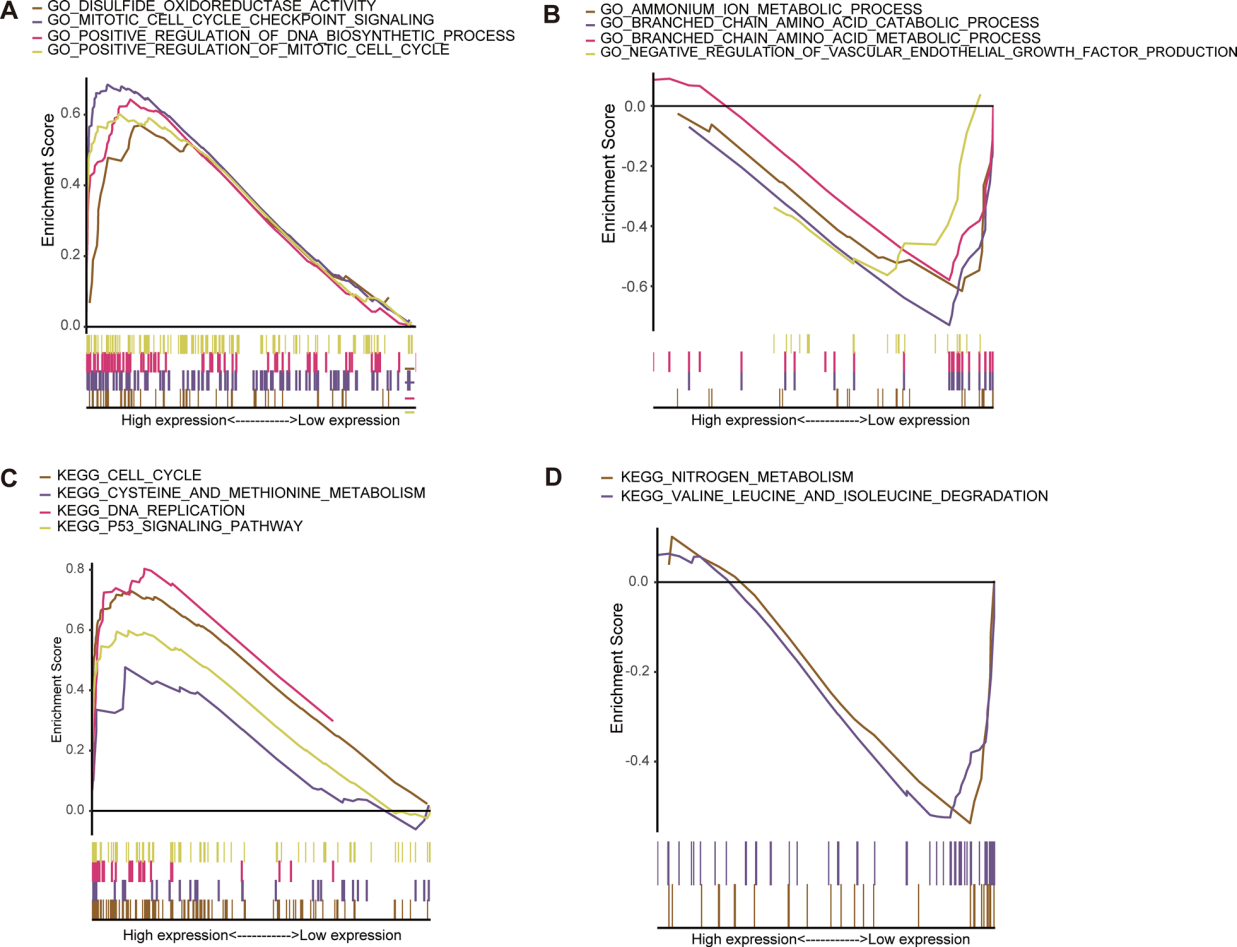
Potential pathway analysis through gene set enrichment analysis. GO enrichment in high-risk group (**A**) and low-risk group (**B**). KEGG enrichment in high-risk group (**C**) and low-risk group (**D**)

In order to get better understand of the potential differences in biological function and pathway between the two risk groups, GSEA was conducted. GO analysis results showed that the regulation of cell cycle and disulfide oxidoreductase activity were enriched in the high-risk group (Fig. 6A). Multiple branched chain amino acid metabolism and negative regulatory pathways of vascular endothelial growth factor were considerably enriched in the low-risk group (Fig. 6B). KEGG results showed that cysteine and methionine metabolic pathways and cell cycle were activated in the high-risk group (Fig. 6C), while nitrogen metabolism and branched chain amino acid metabolism were activated in the low-risk group (Fig. 6D). Overall, in the high-risk group, the pathways that promote tumor growth were mainly activated. The intake of cysteine and methionine can activate the ferroptosis pathway in glioma mouse models, thereby inhibiting tumor growth [21]. The metabolism of branched chain amino acids was an important nitrogen source for tumor cells. Supplementing with branched chain amino acids in the diet can inhibit tumor growth and postoperative recurrence. But controlling the intake of amino acids through diet still requires comprehensive investigation [22].

### Characteristics of tumor microenvironment

**Fig. 7:**
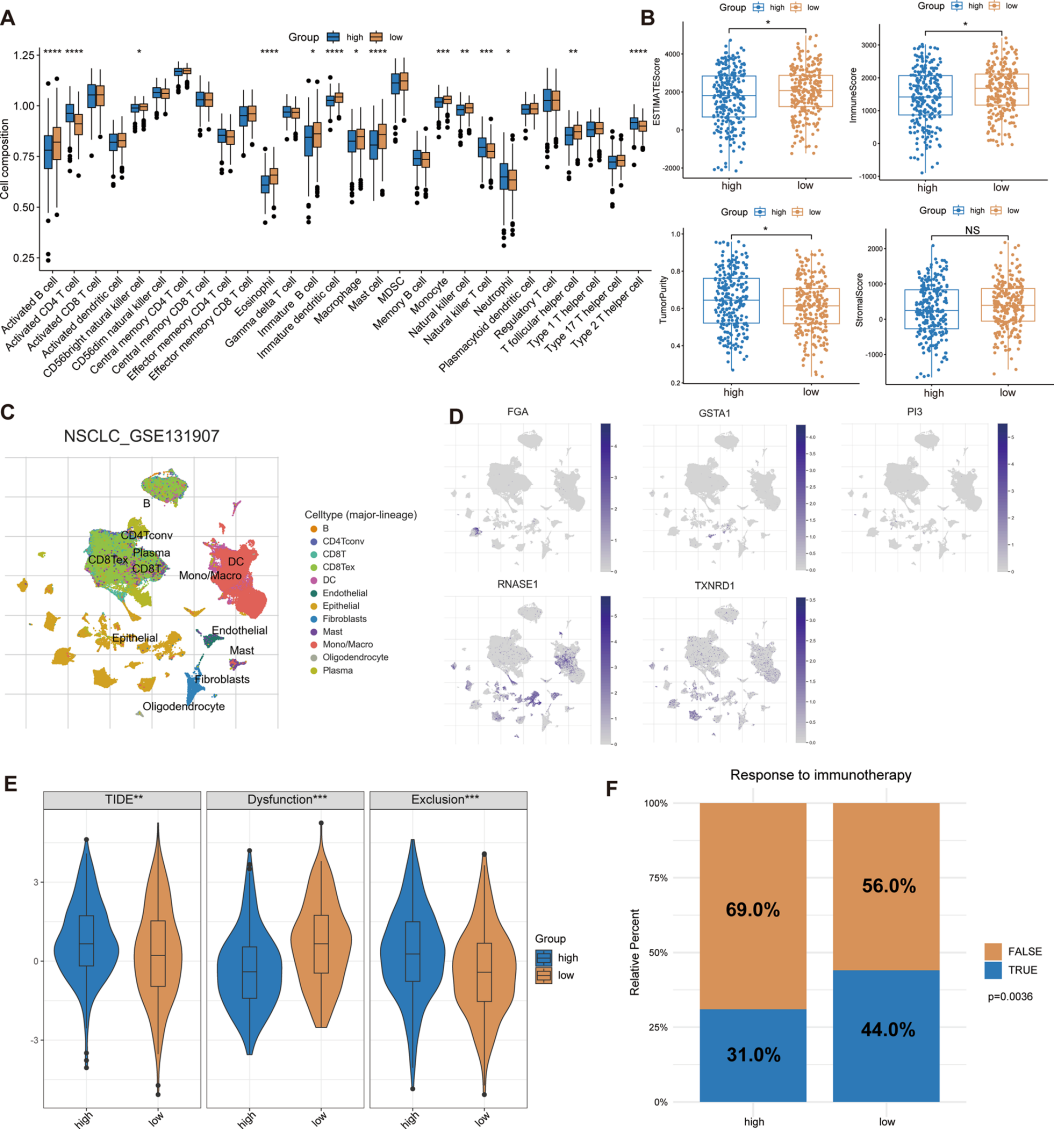
Tumor microenvironment assessment and prediction of immunotherapy response. **A** Comparison of 28 types of immune cell infiltration levels. **B** ESTIMATE algorithm for evaluating tumor microenvironment, including ESTIMATE score, immune score, stromal score, and tumor purity. **C** Display the distribution of different types cells in scRNA level using dimensionality reduction clustering. **D** The distribution of 5 key genes in scRNA level. **E** The difference of TIDE score. **F** Prediction of response to immunotherapy through TIDE algorithm. *p < 0.05, **p < 0.01, ***p < 0.001, ****p < 0.0001

First, we implemented the ssGSEA algorithm to check out the relative proportions of 28 different types of immune cells in TME (Fig. 7A), and found there were notable changes in components in TME components between different risk groups. In the low-risk group, the infiltration of B cells, eosinophils, mast cell, dendritic cells and NK cells was higher. While in the high-risk group, the proportion of CD4 T cells, neutrophils, and Th2 cells is higher. According to the results of ESTIMATE, the high-risk group had higher tumor purity whereas the low-risk group had higher ESTIMATE score and immunological score. No discernible difference existed between the two categories in terms of stromal score. (Fig. 7B). This provides additional confirmation that greater immune cell infiltration in the TME is one factor contributing to the better outcome in the low-risk group. Subsequently, we analyzed the expression of five key genes in LUAD at the single-cell level by using the TISCH database (Fig. 7C). *FGA*, *GSTA1*, and *PI3* were chiefly distributed in epithelial cells, *RNASE1* was chiefly distributed in endothelial cells, monocytes/macrophage, dendritic cells, *TXNRD1* was chiefly distributed in epidermal cells, and DC, monocyte/macrophage, and fibroblasts (Fig. 7D).

### Prediction of immunotherapy response

We examined the variations in immunotherapy response between two risk groups using the TIDE algorithm. The findings revealed that the high-risk group had higher TIDE and exclusion scores whereas the low-risk group had higher dysfunction scores (Fig. 7E). This means that high-risk groups were more likely to experience immune escape when receiving immunotherapy. In addition, we predicted the proportion of people who responded to immunotherapy and we discovered that the low-risk group would see a greater rate of response (chi-square test, p = 0.0036, Fig. 7F). The dataset treated with anti-PD-1 immunotherapy indicated that the high-risk group had a worse prognosis than the low-risk group (Additional file 2: Fig. S2A), which indicated that the riskscore could effectively predict the prognosis of patients receiving PD-1 treatment.

### Silencing *TXNRD1* inhibited LUAD proliferation, migration, and invasion in cell lines

**Fig. 8:**
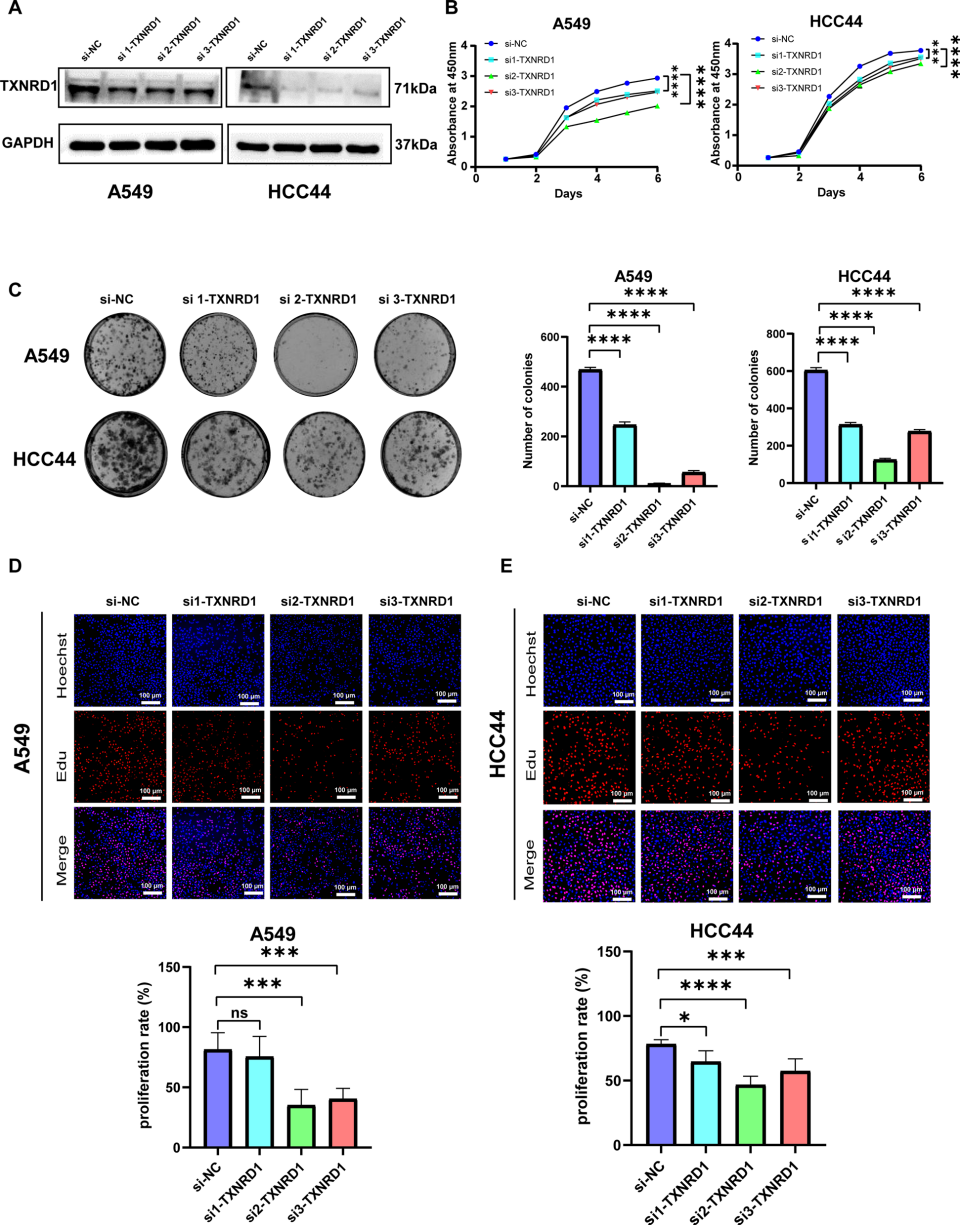
Knocking down *TXNRD1* inhibits the proliferation of LUAD in vitro experiments. **A** Western blotting confirms that *TXNRD1* expression decreases after knockdown. **B** The CCK8 proliferation experiment showed that inhibiting the expression of *TXNRD1* would inhibit the proliferation of LUAD. **C** Plate cloning indicates significant inhibition of clone formation ability after knocking down *TXNRD1*. **D** EDU experiments showed a significant decrease in the proportion of LUAD cells in proliferative state after *TXNRD1* knockdown

**Fig. 9:**
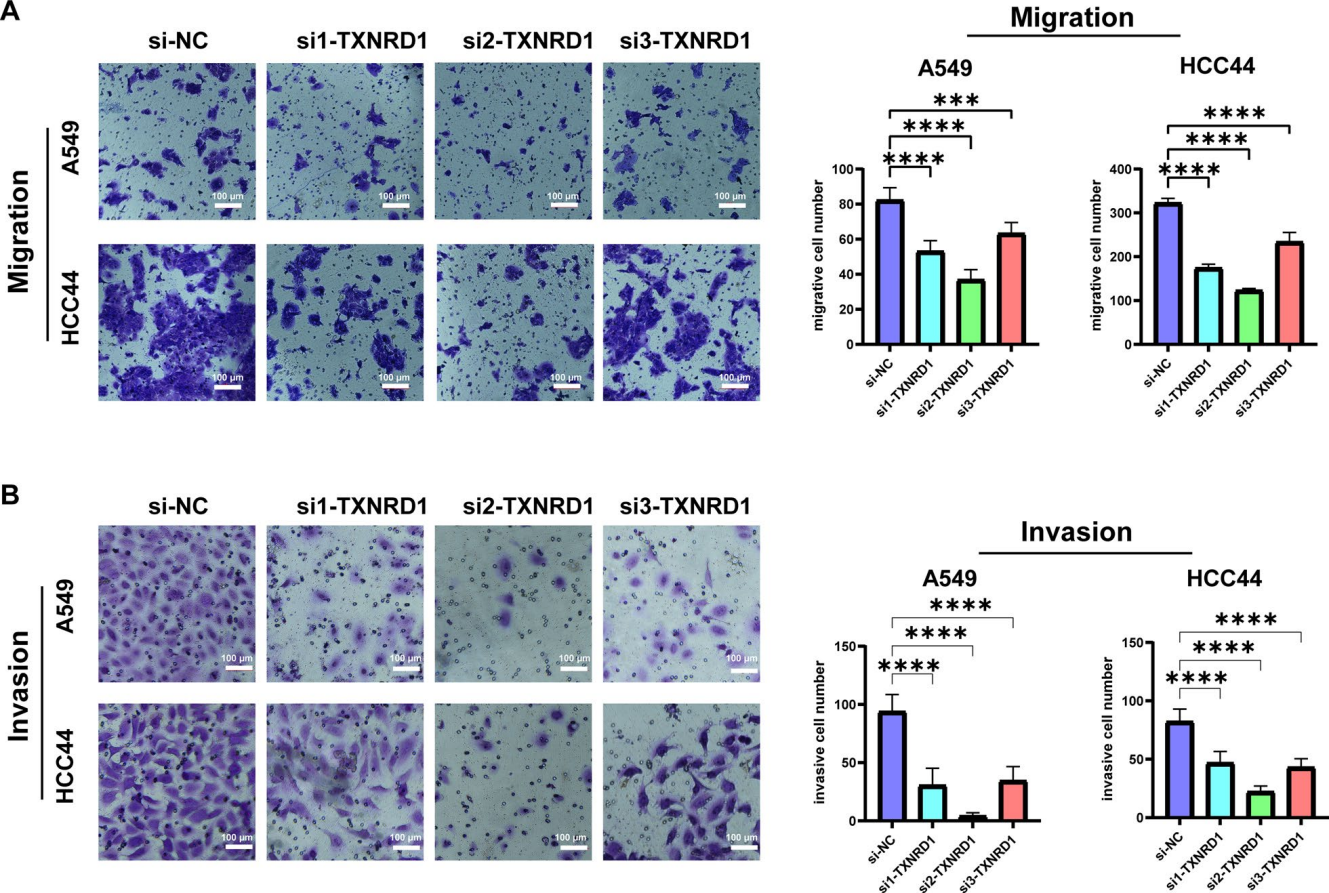
Knocking down *TXNRD1* inhibits the migration and invasion ability of LUAD cells. The migration (**A**) and invasion (**B**) ability of LUAD cells decreased significantly after *TXNRD1* knockdown

Western blotting confirmed that *TXNRD1* was successfully knocked down in A549 and HCC44 (Fig. 8A). Then, we demonstrated through various methods that *TXNRD1* affects the proliferation of LUAD cells. In CCK8, plate clone formation and Edu assays, the interference of *TXNRD1* resulted in significant inhibition of A549 and HCC44 cell viability and proliferation (Fig. 8B–E). Moreover, the suppression of *TXNRD1* resulted in a significant reduction in the migration and invasion abilities of LUAD cells (Fig. 9A, B). These findings strongly indicate that *TXNRD1* may give advantage to LUAD proliferation, migration, and invasion.

## Discussion

As the lung cancer with the highest incidence rate and mortality in the world, most patients have no obvious symptoms of discomfort in the early stage. And by the time the symptoms of discomfort appear, it is often terminal. Traditional chemotherapy can only improve the prognosis of NSCLC by 5%, and the emergence of targeted therapy and immunotherapy has greatly improved the survival status of patients. Especially with the emergence of immunotherapy, it has revolutionized the treatment mode of NSCLC.

In recent years, a variety of cell death modes have been confirmed to widely affect the progression of tumors [23, 24]. Liu et al. found that cancer cells with high *SLC7A11* expression promote NADPH depletion and thus trigger disulfide stress [8]. The proteins related to the formation of disulfide bonds are mainly involved in the formation of actin cytoskeleton. The formation of excessive disulfide bonds leads to the abnormal cross-linking of disulfide bonds, which ultimately leads to the destruction of cell structure and death. Inhibiting the continuous accumulation of disulfide bonds, that is, targeting disulfide metabolism, may be a novel approach to cancer therapy. Although research into disulfide death has only just begun, there is reason to believe that it could be one of the key tools to overcome cancer in the future.

With the continuous progress of sequencing methods, the construction of tumor related prognostic models using transcriptomic data has been recognized by researchers [25–27]. In this study, we analyzed the characteristics of disulfide metabolism related genes in LUAD patients with different expression levels of *SLC711A* and constructed a corresponding risk index, which can effectively identify the prognosis of LUAD patients. In addition, cox regression analysis has shown that it is an independent risk factor for the prognosis of LUAD patients. By combining clinical features to construct nomogram, its practicality had been increased. The GSEA analysis results indicate that the high-risk group was mainly related to the cell proliferation process and the metabolism of sulfur-containing amino acids, while the low-risk group was mainly related to the branched chain amino acids metabolism. Yue et al. found through in vitro and in vivo experiments that limiting the intake of sulfur-containing amino acids significantly inhibited the growth of colon cancer cells in vivo, and had a good synergistic effect with PD-L1 inhibitors [28]. This may be one of the reasons that affects the prognosis of high-risk group patients.

Our study identified five key genes involved in disulfide metabolism. Fibrinogen α (*FGA*), which is involved in the formation of extracellular matrix proteins, promotes cell proliferation, migration and invasion through CRISPR/Cas9 knockdown of *FGA* in LUAD, and might turn into a new therapeutic target [29]. *GSTA1* plays an important role in regulating glutathione metabolism. In lung cancer, overexpression of *GSTA1* plays a role in tumor promotion [30]. *PI3* (Peptidase Inhibitor 3) is a serine protease inhibitor which is involved in breast cancer. High expression of *PI3* is significantly associated with poor prognosis of high-grade serous ovarian and breast cancer, and in vitro and in vivo studies have demonstrated that overexpression of *PI3* promotes the proliferation of breast cancer [31, 32]. *RNASE1* encodes endonuclease, which regulates extracellular RNA clearance and immune function. Li et al. found that *RNASE1* promotes breast cancer by binding to and activating tyrosine kinase receptors [33]. *TXNRD1* comes from the TXNRD family of enzymes, which together with glutathione maintain cell REDOX balance [34]. It is used as a prognostic marker in many cancers [35]. In addition, *TXNRD1* is involved in iron death resistance of cancer cells [36]. Targeting *TXNRD1* to promote cancer cell death due to oxidative stress disorder has the potential to be a new target for cancer therapy. The results of our external experiments have also indicated the multiple malignant oncological behaviors of LUAD affected by *TXNRD1*. Xiong et al. synthesized a new nano-modulator using key genes that can activate oxidative stress-induced cell death, effectively inhibiting tumor growth and eradicating cancer stem cells to suppress lung metastasis [37]. This provides us with new insights. Given the involvement of *TXNRD1* in maintaining redox stability, constructing new nano antibodies to regulate oxidative stress processes in tumors may become a new clinical translational pathway.

The tumor microenvironment's makeup largely influences how well immunotherapy works. [38]. Our research results show that the TME of the two subgroups has different immune landscapes. The low-risk group has a higher level of immune cell infiltration and a significantly better prognosis than the high-risk group. In addition, in the prediction of immune therapy response, the low-risk group had a better proportion of responses, and the difference was statistically significant. Usually, we believe that higher levels of immune cell infiltration lead to better immune therapy responses, which is consistent with our results.

Our study has the following shortcomings. First, our risk index requires large and multicenter clinical trials to be validated. Secondly, the mechanism of how *TXNRD1* regulates ferroptosis needs further study. Finally, the clinical transformation of *TXNRD1* still needs to be confirmed in vivo.

## Conclusion

In summary, we analyzed the characteristics of disulfide metabolism-related genes and constructed a characteristic risk index, which can effectively predict the prognosis of LUAD patients. The in vitro experiments indicate that *TXNRD1* is a potential therapeutic target for LUAD.

### Supplementary Information

**Additional file 1: Figure S1.** **A** KM survival analysis in GSE68465. **B** Changes in the number of deaths as the risk score increases in GSE68465. **C** Heatmap showing the expression in GSE68465. **D** KM survival analysis in GSE37745. **E** Changes in the number of deaths as the risk score increases in GSE37745. **F** Heatmap showing the expression in GSE37745.

**Additional file 2: Figure S2.** **A** KM survival analysis based on the riskscore in anti-PD-1 immunotherapy cohort.

## Data Availability

The transcriptome data involved in this study have been annotated with their sources, and the resulting code can be obtained from the corresponding authors with reasonable requirements.

## References

[1] Siegel RL, Miller KD, Jemal A (2020). Cancer statistics, 2020. CA Cancer J Clin.

[2] Hirsch FR, Scagliotti GV, Mulshine JL, Kwon R, Curran WJ, Wu YL, Paz-Ares L (2017). Lung cancer: current therapies and new targeted treatments. Lancet.

[3] Zappa C, Mousa SA (2016). Non-small cell lung cancer: current treatment and future advances. Transl Lung Cancer Res.

[4] Miller M, Hanna N (2021). Advances in systemic therapy for non-small cell lung cancer. BMJ.

[5] Minguet J, Smith KH, Bramlage P (2016). Targeted therapies for treatment of non-small cell lung cancer—recent advances and future perspectives. Int J Cancer.

[6] Mamdani H, Matosevic S, Khalid AB, Durm G, Jalal SI (2022). Immunotherapy in lung cancer: current landscape and future directions. Front Immunol.

[7] Goji T, Takahara K, Negishi M, Katoh H (2017). Cystine uptake through the cystine/glutamate antiporter xCT triggers glioblastoma cell death under glucose deprivation. J Biol Chem.

[8] Liu X, Nie L, Zhang Y, Yan Y, Wang C, Colic M, Olszewski K, Horbath A, Chen X, Lei G (2023). Actin cytoskeleton vulnerability to disulfide stress mediates disulfidptosis. Nat Cell Biol.

[9] Rebhan M, Chalifa-Caspi V, Prilusky J, Lancet D (1997). GeneCards: integrating information about genes, proteins and diseases. Trends Genet.

[10] Ritchie ME, Phipson B, Wu D, Hu Y, Law CW, Shi W, Smyth GK (2015). limma powers differential expression analyses for RNA-sequencing and microarray studies. Nucleic Acids Res.

[11] Yu G, Wang LG, Han Y, He QY (2012). clusterProfiler: an R package for comparing biological themes among gene clusters. OMICS.

[12] Mayakonda A, Lin DC, Assenov Y, Plass C, Koeffler HP (2018). Maftools: efficient and comprehensive analysis of somatic variants in cancer. Genome Res.

[13] Subramanian A, Tamayo P, Mootha VK, Mukherjee S, Ebert BL, Gillette MA, Paulovich A, Pomeroy SL, Golub TR, Lander ES (2005). Gene set enrichment analysis: a knowledge-based approach for interpreting genome-wide expression profiles. Proc Natl Acad Sci USA.

[14] Barbie DA, Tamayo P, Boehm JS, Kim SY, Moody SE, Dunn IF, Schinzel AC, Sandy P, Meylan E, Scholl C (2009). Systematic RNA interference reveals that oncogenic KRAS-driven cancers require TBK1. Nature.

[15] Yoshihara K, Shahmoradgoli M, Martinez E, Vegesna R, Kim H, Torres-Garcia W, Trevino V, Shen H, Laird PW, Levine DA (2013). Inferring tumour purity and stromal and immune cell admixture from expression data. Nat Commun.

[16] Tirosh I, Venteicher AS, Hebert C, Escalante LE, Patel AP, Yizhak K, Fisher JM, Rodman C, Mount C, Filbin MG (2016). Single-cell RNA-seq supports a developmental hierarchy in human oligodendroglioma. Nature.

[17] Jiang P, Gu S, Pan D, Fu J, Sahu A, Hu X, Li Z, Traugh N, Bu X, Li B (2018). Signatures of T cell dysfunction and exclusion predict cancer immunotherapy response. Nat Med.

[18] Braun DA, Hou Y, Bakouny Z, Ficial M, Sant' Angelo M, Forman J, Ross-Macdonald P, Berger AC, Jegede OA, Elagina L (2020). Interplay of somatic alterations and immune infiltration modulates response to PD-1 blockade in advanced clear cell renal cell carcinoma. Nat Med.

[19] Ren H, Zhang Z, Cheng X, Zou Z, Chen X, He C (2023). Injectable, self-healing hydrogel adhesives with firm tissue adhesion and on-demand biodegradation for sutureless wound closure. Sci Adv.

[20] Zhou W, Lin Z, Xiong Y, Xue H, Song W, Yu T, Chen L, Hu Y, Panayi AC, Sun Y (2021). Dual-targeted nanoplatform regulating the bone immune microenvironment enhances fracture healing. ACS Appl Mater Interfaces.

[21] Upadhyayula PS, Higgins DM, Mela A, Banu M, Dovas A, Zandkarimi F, Patel P, Mahajan A, Humala N, Nguyen TTT (2023). Dietary restriction of cysteine and methionine sensitizes gliomas to ferroptosis and induces alterations in energetic metabolism. Nat Commun.

[22] Peng H, Wang Y, Luo W (2020). Multifaceted role of branched-chain amino acid metabolism in cancer. Oncogene.

[23] Bertheloot D, Latz E, Franklin BS (2021). Necroptosis, pyroptosis and apoptosis: an intricate game of cell death. Cell Mol Immunol.

[24] Hanggi K, Ruffell B (2023). Cell death, therapeutics, and the immune response in cancer. Trends Cancer.

[25] Hong M, Tao S, Zhang L, Diao LT, Huang X, Huang S, Xie SJ, Xiao ZD, Zhang H (2020). RNA sequencing: new technologies and applications in cancer research. J Hematol Oncol.

[26] Guo CR, Mao Y, Jiang F, Juan CX, Zhou GP, Li N (2022). Computational detection of a genome instability-derived lncRNA signature for predicting the clinical outcome of lung adenocarcinoma. Cancer Med.

[27] Zhang W, Qu H, Ma X, Li L, Wei Y, Wang Y, Zeng R, Nie Y, Zhang C, Yin K (2023). Identification of cuproptosis and immune-related gene prognostic signature in lung adenocarcinoma. Front Immunol.

[28] Yue T, Li J, Zhu J, Zuo S, Wang X, Liu Y, Liu J, Liu X, Wang P, Chen S (2023). Hydrogen sulfide creates a favorable immune microenvironment for colon cancer. Cancer Res.

[29] Wang M, Zhang G, Zhang Y, Cui X, Wang S, Gao S, Wang Y, Liu Y, Bae JH, Yang WH (2020). Fibrinogen alpha chain knockout promotes tumor growth and metastasis through integrin-AKT signaling pathway in lung cancer. Mol Cancer Res.

[30] Liu H, Yang Z, Zang L, Wang G, Zhou S, Jin G, Yang Z, Pan X (2018). Downregulation of glutathione S-transferase A1 suppressed tumor growth and induced cell apoptosis in A549 cell line. Oncol Lett.

[31] Labidi-Galy SI, Clauss A, Ng V, Duraisamy S, Elias KM, Piao HY, Bilal E, Davidowitz RA, Lu Y, Badalian-Very G (2015). Elafin drives poor outcome in high-grade serous ovarian cancers and basal-like breast tumors. Oncogene.

[32] Hunt KK, Wingate H, Yokota T, Liu Y, Mills GB, Zhang F, Fang B, Su CH, Zhang M, Yi M (2013). Elafin, an inhibitor of elastase, is a prognostic indicator in breast cancer. Breast Cancer Res.

[33] Lee HH, Wang YN, Yang WH, Xia W, Wei Y, Chan LC, Wang YH, Jiang Z, Xu S, Yao J (2021). Human ribonuclease 1 serves as a secretory ligand of ephrin A4 receptor and induces breast tumor initiation. Nat Commun.

[34] Gencheva R, Arner ESJ (2022). Thioredoxin reductase inhibition for cancer therapy. Annu Rev Pharmacol Toxicol.

[35] Shimada BK, Swanson S, Toh P, Seale LA (2022). Metabolism of selenium, selenocysteine, and selenoproteins in ferroptosis in solid tumor cancers. Biomolecules.

[36] Zhang J, Li X, Zhao Z, Cai W, Fang J (2023). Thioredoxin signaling pathways in cancer. Antioxid Redox Signal.

[37] Xiong Y, Yong Z, Li S, Wang Q, Chen X, Zhang Z, Zhao Q, Deng Q, Yang X, Li Z (2023). Self-reliant nanomedicine with long-lasting glutathione depletion ability disrupts adaptive redox homeostasis and suppresses cancer stem cells. Adv Funct Mater.

[38] Ngwa VM, Edwards DN, Philip M, Chen J (2019). Microenvironmental metabolism regulates antitumor immunity. Cancer Res.

