# Supplementary figures and images for: Development and validation of a disulfidptosis and disulfide metabolism-related risk index for predicting prognosis in lung adenocarcinoma
Source: Cancer Cell Int. 2024 Jan 2;24:2. doi: 10.1186/s12935-023-03204-1 (PMC10763446; doi:10.1186/s12935-023-03204-1)

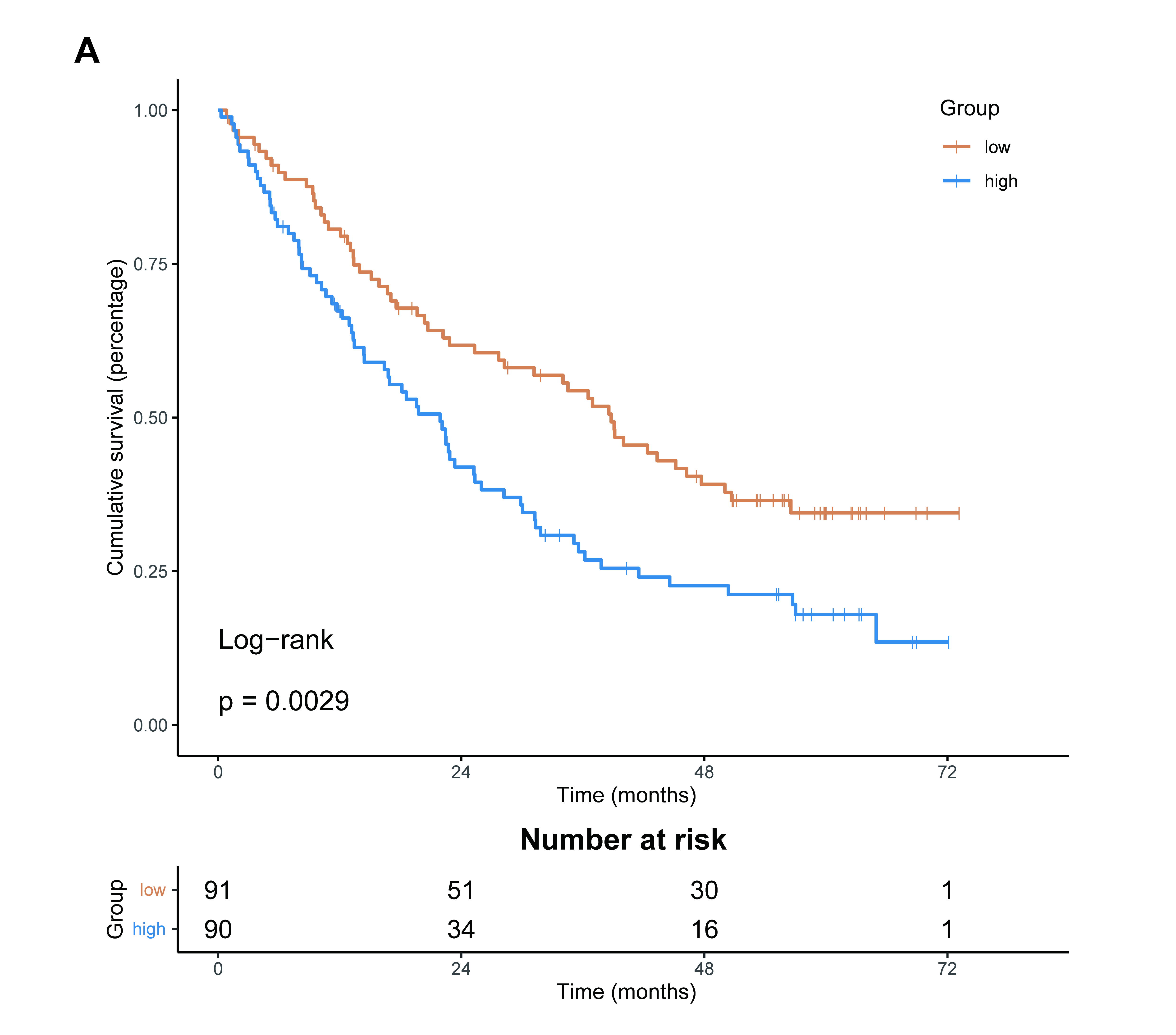

Supplement: Supplementary file 2 — Additional file 2: Figure S2. A KM survival analysis based on the riskscore in anti-PD-1 immunotherapy cohort. [file 12935_2023_3204_MOESM2_ESM.tif]
